# Assessing the Effect of Bilateral Trade on Health in the Asian Region: Does Digitization Matter?

**DOI:** 10.3389/fpubh.2021.802465

**Published:** 2022-01-12

**Authors:** Xinmin Zhang, Xinqin Zhang, Xiao-Guang Yue, Faisal Mustafa

**Affiliations:** ^1^School of Economics and Management, Huanghuai University, Zhumadian, China; ^2^Zhumadian Academy of Industry Innovation and Development, Huanghuai University, Zhumadian, China; ^3^School of Sciences, European University Cyprus, Nicosia, Cyprus; ^4^UCP Business School, Faculty of Management Studies, University of Central Punjab, Lahore, Pakistan

**Keywords:** bilateral trade, digitalization, health, Asian economies, life expectancy

## Abstract

A recurrent theme of the literature and wider public discourse is that trade and digitization are good for health as it promotes economic prosperity. The present study investigates the impact of trade and digitization on health in 12 selected Asian economies for the period 1991–2019. The study applied FMOLS and DOLS approaches for confirming the panel and economy-wise findings. The core findings of the panel FMOLS confirm the significant negative impact of trade and digitization on mortality rate, and trade and digitization have significantly positively contributed to life expectancy in selected Asian countries in the long run. The study deduces some imperative policy implications related to trade, digitization, and health, specifically for Asian economies.

## Introduction

Defining good health is a complex phenomenon because the absence of physical illness is not an indication of good health, rather a person's mind should also be healthy, and they should be an active part of society (1). Health has become the most primary concern for individuals and governments all around the globe. A healthy mind and body can contribute to the well-being of society with much more efficiency and become an asset for society. On the other side, a physically and mentally ill person can become a liability for society. As a result, demand for good health facilities has become a basic necessity alongside food, shelter, and education. Since 1950, the provision of good health services has added 3 years per decade to the life expectancy of people worldwide. However, the situation of health and its related indicators size life expectancy, has not improved in my developing economies. This argument can be fortified because a drastic disparity in the minimum and maximum life expectancy scores can be observed worldwide. The average maximum and minimum life expectancy scores are recorded at about 81.49 and 39.54, respectively. Likewise, the maximum and minimum average infant mortality rates are recorded at about 147.86 and 3.89, respectively. Low health status is becoming a serious problem in the context of universal health, with a growing proportion of the world's people suffering from some sort of physical or mental health issue. Given the consequence of poor health as a vital promoter to disability-adjusted life years (DALYs) and immobilizing conditions, there is an urgent need to comprehend how international and national health strategies and agendas may improve this burden.

In 2015, the WHO and its member countries confirmed to work on trade and health while minimizing risks and maximizing the opportunities to enhance public health (2). Many people around the globe travel to other countries to get medical care, contributing to the budget of the host economy through related medical tourism. Traveling people to other countries to obtain medical facilities and their associated health and goods services can influence the health of the population and the health system. Similarly, new trade negotiations and regional trade agreements exist throughout the world, including Asia, and have risen over time. Generally, foreign trade integration has increased in recent years (3).

Free trade is among the fundamental elements of economic growth in various economies that generate employment, diminish poverty in many economies, increase the level of income, raise competition among organizations, raise foreign exchange, and transfer knowledge (4). It also supports economies to generate exports and imports of goods and services through opening borders for economic flows. Trade globalization can affect the health sector through various channels, either positively or negatively. For instance, policies of trade can influence beverages, nutritional consumption, and pharmaceutical drugs consumption. It is also possible that the consumption of beverages, food items, and tobacco through trade agreements between Europe and Asia can form a macroeconomic structure that can either generate benefits or risks to health. For instance, obesity in children can be associated with foreign trade in terms of food consumption, accumulation of formula for infants, and less nutritional food for children.

Trade globalization can also influence health when people interact with each other and may spread transmittable diseases such as COVID-19 and HIV that create risks to people's health. Correspondingly, pollution generated due to trade directly influences public health (5). For example, highly developed economies used to export harmful materials to lower-income economies; such material contains waste of electronics that discharge toxicants (6). It is estimated that almost 700,000 people have died in Western Europe due to pollution linked with goods imported from China. The high spread of a tropical illness such as malaria in lower-income economies deteriorates health and trade (7). This indicates that traders are not ideal in disease-prevalent areas because these areas can create risks for both health and trade segments. Long-term upsurges in life expectancy are another significant channel in trade globalization (8).

Many economists believe that trade openness enhances well-being by improving average incomes and economic development (9). The people in the health community having lower-income economies are more unconvinced toward the influence of free trade. In this group, trade liberalization is alleged to adversely affect the so-called “determinants of health” such as the environmental, economic, and social conditions that affect the health of populations and individuals (10). In the literature, it is also argued that trade liberalization directly harms people's health by increasing economic insecurity and income inequality, by raising the accessibility of harmful processed foods, and by damaging the environment (11, 12). Academics of public health usually condemn bilateral and multilateral free trade agreements as encouraging a “neoliberal agenda” that enforces obligatory privatizations of health services and improperly stringent standards of rational property protection on the poor people (13). These arguments highlight the harmful health influences of increased trade openness, of which globalization of trade is an imperative determinant.

Over the last three decades, a noteworthy development in information and communication technologies (ICTs) has designed new sources of invention and knowledge diffusion. ICT can serve as a catalyst in increasing the productivity of many industries, promoting economic growth (14). Among the different proxies of ICT, the most vital is the internet that can help to connect billions of people and is considered a key source to diffuse knowledge (15). In this context, a report published by OECD (16) is worth mentioning, and it states that “the internet, as a connector on a massive scale, provides the opportunity to access and share knowledge in ways that were previously not possible.” The primary feature of the internet is that it has improved the ability to promote invention and information at a universal level at a much higher speed than expected. As a result of this, it is more likely that knowledge will become a proxy of the international public good.

At the end of the previous century, when the internet started to develop at a rapid pace, the process of innovation and knowledge diffusion also grew rapidly OECD (16), empowering “less-developed countries to tap into the global knowledge pool, a larger knowledge pool than they ever had access to before” (17). During the period 1990–2014, global internet users swelled from 394 million to 1.858 billion (18), and these numbers came from both developed and developing economies. The foremost advantage of the internet can be seen in developing economies where the internet has reduced the time required by developing economies to diffuse knowledge in their production process due to the availability of technology that is fast and reliable and can spread knowledge at a much higher speed. This has facilitated these nations to decrease replication costs as followers of the invention spread, manage, and generate knowledge, intermingling with the leaders of inventions and enlightening the circumstances for inventions (17). To sum up this discussion, we can say that the internet has been influential in taming the “innovation sharing processes” among nations (19).

Given the fact that ICT can help promote the process of knowledge diffusion and can contribute to economic production, which in turn speed up the process of economic development, we can reckon that it can also improve health outcomes, which are a by-product of economic well-being. However, whether the improved availability of information and communication technologies (ICTs) can promote health outcomes or not is a question that needs to be adequately answered. In a bid to answer this question, a debate has erupted among the ranks of economic policymakers and health officials on the positive outcomes of ICT on health status. Consistent with this view, there has been substantial development in internet availability in cities and metropolitans. According to Bukachi and Pakenham-Walsh (20), staff working in hospitals and health care units can utilize the easy accessibility and availability of the internet as a source of communication, information related to health care programs, and worldwide association.

In the past, several pieces of theoretical and empirical evidence contended that ICT could bring a positive change in the health sector of developing economies and thus improve public health (21). The internet is a source of communication and can provide necessary health care services, such as services to pregnant women. Another important advantage of the internet is that it can speed up the communication process between patients and health care staff (22). Moreover, it is sources that can diffuse the ever-changing medical knowledge and research from developed to developing nations quickly. Information and communication technology also supports the people to raise the level of awareness with regards to the advantages of good health and disadvantages of bad health. For example, the internet offers the public access to almost infinite information related to their health issues and by gathering all the available information even a layman can judge their symptoms with ease.

Despite the observance of the positive role of trade and ICT in improving health status in a few instances, the literature is still not large enough. Hence, in this study, we try to plug this gap in the literature, and our primary concern is to see the impact of trade and digitalization on health outcomes in Asian economies. This is the first-ever study that has tried to analyze the nexus between trade, digitalization, and health outcomes to the best of our knowledge. The core findings of our research will provide some important policy implications, mainly for Asian countries. The study highlights the health outcomes from the development of trade and digitalization and a policy that values sustainable development. In section Model and Methods, we provided the details about data and methodology, followed by the results in section Results and Discussion and the conclusion in section Conclusion and Policy Implication.

## Model and Methods

To achieve the objective, we have borrowed a theoretical model from Bergh and Nilsson (23) and Whiting and Williams (24) and empirical models followed by Dutta et al. (25) and Byaro et al. (26). The basic form is:


$$\begin{array}{l} \text{Healt}{\text{h}}_{\text{ it}}=\;{\text{φ}}_{1}+\;\varphi_{1}\text{Trad}{\text{e}}_{\text{it}}+{\text{φ}}_{2}\text{Interne}{\text{t}}_{\text{it}}+{\text{φ}}_{3}\text{GD}{\text{P}}_{\text{it}}\\ +\;{\text{φ}}_{4}\text{H}{\text{E}}_{\text{it}}+{\text{ε}}_{\text{it}} \end{array}$$

Health, Trade, Internet, GDP, and HE represent infant mortality and life expectancy, trade openness, internet users, GDP per capita, and health expenditure, respectively. The countries are denoted by the subscript i(i=1, ......, 12), while t signifies the data period (t=1, ......., 29). In model, φ_1_, φ_2_, φ_3_, and φ_4_ are the elasticities of health outcomes with respect to trade openness, internet users, GDP per capita, and health expenditure, while α_*i*_ represents unobservable individual effects, and εit is the error term in the model. The extended form is:


$$\begin{array}{l} \text{Healt}{\text{h}}_{\text{ it}}=\;{\text{φ}}_{1}+\;{\text{φ}}_{1}\text{Trad}{\text{e}}_{\text{it}}+{\text{φ}}_{2}\text{Interne}{\text{t}}_{\text{it}}+{\text{φ}}_{3}\text{GD}{\text{P}}_{\text{it}}\\ +\;{\text{φ}}_{4}\text{H}{\text{E}}_{\text{it}}+{\text{α}}_{\text{i}}+{\text{ε}}_{\text{it}} \end{array}$$

The data used in the analysis is based on panel settings which have many advantages over cross-sectional and time-series data. It combines the observations of various cross-sectional units across time. Therefore, the number of observations increased, and the problem of degree of freedom was also resolved. Moreover, the panel data provide more variability, more information, and a higher degree of efficiency in a data set than simple time-series and cross-sectional data (27). Accordingly, panel data techniques are used to get the estimates of the variables. However, the basic panel data techniques such as fixed effect (FE), random effect (RE), and generalized methods of movement (GMM) are appropriate when T is small, and N is large. Whereas, when T is large, we need to apply panel cointegration methods. Hence, to investigate the impact of bilateral trade on health outcomes in the Asian region, we have taken recourse panel data techniques such as pooled ordinary least square (POLS), fully modified ordinary least square (FMOLS), and dynamic ordinary square (DOLS).

The first method we use in the analysis is POLS, which combines all the observations and then estimates them in a single regression by ignoring their time-series and cross-sectional behavior. Pooled OLS is a good technique for employing a different sample in panel data (28). However, in POLS, the results are inconsistent due to the problem of unobserved heterogeneity because POLS cannot account for it (29). Subsequently, the study used FMOLS and DOLS, which are very efficient estimators in the presence of endogeneity among independent variables, and can also manage the problem of serial correlation in the error terms. To control endogeneity and serial correlation, the method used by FMOLS is non-parametric, whereas DOLS include the lead as well as the lag values of the independent variables, which is the parametric approach as opposed to non-parametric, to control the problems of endogeneity and serial correlation (30). Another advantage of DOLS is that it can also produce efficient results even if the size of a sample is small (31). Further, in the case of cross-sectional dependence, which is a serious problem in panel data, DOLS is an efficient method and provides consistent and unbiased results. To handle cross-sectional dependence, the DOLS relies on obtaining the country-specific coefficients, which help to achieve unbiased and consistent estimators. In the panel data, another problem is the problem of heterogeneity in the long-run variance, and cointegrated panel and FMOLS and DOLS with the help of their weighted criteria can also control the problem of heterogeneity.

### Data

The core objective of the study is to examine the impact of trade and digitization on the health of the population in selected 12 Asian economies: China, India, Japan, Indonesia, Republic of Korea, Saudi Arabia, Iran, Turkey, Thailand, Malaysia, Singapore, and the Philippines. The study used life expectancy at birth and infant mortality rate to measure the health of the population (infant mortality is measured per 1,000 live births). We used trade and digitization as our independent variables in empirical analysis. Trade openness can affect health through economic growth and technology transfer (26). ICT can improve health outcomes by reducing poverty alleviation, geographical constraints and improving knowledge diffusion and economic development (25). The trade variable is measured via imports and exports, and digitization is measured through the internet. The trade variable is measured as a proportion of imports and exports to GDP. The internet variable is measured as the proportion of internet users in the population. Along with these focus variables, the study incorporated the role of GDP and health expenditure as control variables for inferring the impact of trade and digitization on health. GDP is taken as constant 2010 US$, and health expenditure is measured as a proportion of GDP. All selected control variables are important for health. For performing the empirical task, the data on all these variables are taken from the World Bank. The mean of IM, LE, trade, internet, GDP, and HE are 20.25 per live birth, 73.06, 4.217%, 23.90%, 8.876 US$, and 4.219%, respectively. The details of variables are reported in Table 1.

**Table 1:** Definitions and data sources.

**Variables**	**Symbol**	**Variable**	**Mean**	**Std. Dev**.	**Sources**
Infant Mortality	IM	Mortality rate, infant (per 1,000 live births)	20.252	17.395	World Bank
Life expectancy	LE	Life expectancy at birth, total (years)	73.069	5.425	World Bank
Trade openness	Trade	Trade (% of GDP)	4.217	0.736	World Bank
Internet users	Internet	Internet users (% of population)	23.900	28.200	World Bank
Gross domestic product	GDP	GDP per capita (constant 2010 US$)	8.876	1.183	World Bank
Health expenditure	HE	Current health expenditure (% of GDP)	4.219	1.828	World Bank

## Results and Discussion

Before commencement of regressions analysis, it is necessary to confirm that all the series either contain or do not contain cross-sectional dependence. Economies are interdependent on each other due to developments of various economic policies, such as health policies, regional economic policies, energy policies, financial policies, environmental policies, and foreign trade policies, as well as their dissimilarities in stages of development, (underdeveloped, emerging, or developed economies). This discloses the existence of cross-sectional dependence among economies. The study applied a cross-sectional dependence test developed by Pesaran (32). In the presence of cross-sectional dependence, conventional panel unit root tests provide insignificant and ineffective results. In this regard, the applied IPS and LLC methods for the purpose of panel unit root testing. Table 2 displays the outcomes of the cross-sectional dependence test, and Table 3 shows the findings of panel unit root and cointegration tests. The empirical results of the cross-sectional dependence test in Table 2 reveal that the null hypothesis of the cross-sectional dependence is not accepted, while the alternative hypothesis of the test is strongly accepted, as shown in Table 3.

**Table 2:** Cross-sectional dependence and cointegration test.

	**Cross-sectional dependence**				
	**IM**	**Trade**	**GDP**	**HE**	**Internet**
Pesaran's test	5.069^***^	6.240^***^	1.918^**^	0.756	1.057
Off-diagonal elements	0.439	0.382	0.677	0.538	0.422
	**LE**	**Trade**	**GDP**	**HE**	**Phy**
Pesaran's test	6.542	6.784	2.242^**^	0.722	1.078
Off-diagonal elements	0.412	0.383	0.621	0.556	0.438

*** *p < 0.01, and*

** *p < 0.05*.

**Table 3:** Panel unit root testing.

	**LLC**			**IPS**		
	**I(0)**	**I(1)**	**Decision**	**I(0)**	**I(1)**	**Decision**
**Panel unit root**						
IM	−0.563	−5.537^***^	I(1)	−1.369	−4.298^***^	I(1)
LE	−0.421	−6.759^***^	I(1)	−1.502	−5.365^***^	I(1)
Trade	1.235	−1.409^*^	I(1)	−1.645	−2.107^***^	I(1)
GDP	−0.318	−6.723^***^	I(1)	−1.454	−3.492^***^	I(1)
HE	−0.565	−6.775^***^	I(1)	−0.755	−3.814^***^	I(1)
Internet	1.871	−2.039^**^	I(1)	−0.711	−3.751^***^	I(1)
**Cointegration test**						
**IM-model**						
DHg	8.321^***^					
DHp	8.545^***^					
**LE-model**						
DHg	7.985^***^					
DHp	8.356^***^					

*** *p < 0.01*,

** *p < 0.05, and*

* *p < 0.1*.

Findings strongly nullified the null hypothesis of the unit root tests at the level and accepted the alternative hypothesis of the unit root test. These findings confirm that all the variables are stationary at the first difference, i.e., I(1) stationary. The concern variables have a long-run relationship in selected Asian countries for the period of 1991–2019. The long-run impacts of bilateral trade and digitalization on public health are measured by applying the FMOLS approach and for confirming the robustness of findings. The study measured public health through two proxies, infant mortality rate and life expectancy. Table 4 shows the group-wise findings of FMOLS and DOLS approaches for both models, and Table 5 shows economy-wise result estimates of FMOLS, and DOLS approaches for both models.

**Table 4:** Panel estimates of FMOLS and DOLS.

	**FMOLS**				**DOLS**			
	**IM**		**LE**		**IM**		**LE**	
	**beta**	**t-stat**	**beta**	**t-stat**	**beta**	**t-stat**	**beta**	**t-stat**
Trade	−2.000^***^	(5.481)	0.670^***^	(3.380)	−2.060^***^	(5.570)	1.190^***^	(3.570)
Internet	−2.980^***^	(3.383)	2.000^***^	(4.820)	−2.300^***^	(3.880)	3.590^***^	(7.410)
GDP	−3.352^***^	(7.612)	3.130^***^	(6.020)	−3.980^***^	(6.220)	0.800^***^	(4.200)
HE	−2.140^***^	(8.610)	0.850^***^	(3.890)	−2.550^***^	(7.790)	0.740	(0.230)

*** *p < 0.01*.

**Table 5:** Economy-wise estimates of FMOLS.

	**IM**				**LE**			
	**Trade**	**Internet**	**GDP**	**HE**	**Trade**	**Internet**	**GDP**	**HE**
China	−6.33^***^	−0.43^**^	−5.42^***^	−0.75^***^	0.44^***^	0.43^***^	3.03^***^	0.46^***^
	(3.79)	(2.43)	(6.14)	(6.34)	(11.9)	(2.78)	(9.45)	(2.89)
India	−9.14^***^	−0.09	−4.26^***^	−4.71^***^	2.16^***^	1.30	7.12^***^	1.08^***^
	(5.38)	(0.04)	(7.09)	(7.48)	(7.62)	(0.68)	(10.5)	(3.35)
Japan	−1.01^***^	−2.41^***^	1.85^**^	−0.07^***^	1.85^***^	6.48^***^	−2.75^***^	−0.05
	(8.04)	(7.24)	(2.17)	(3.01)	(8.94)	(11.7)	(1.95)	(1.33)
Indonesia	−3.28^***^	−2.99^***^	−3.01^***^	−1.63^***^	1.89^***^	1.51^**^	8.15^***^	1.89^***^
	(7.21)	(2.66)	(7.71)	(8.94)	(7.66)	(2.31)	(5.13)	(3.89)
Turkey	−3.45^***^	−5.41^***^	5.75^***^	−1.03^**^	0.51^**^	2.72^***^	0.95^***^	−0.22^**^
	(3.41)	(8.22)	(3.91)	(2.49)	(2.41)	(4.77)	(3.13)	(2.31)
Korea, Rep.	−1.16^***^	−0.09	−3.71^***^	0.76^***^	0.04	0.37^***^	3.32^***^	1.35^***^
	(3.87)	(0.79)	(5.46)	(5.93)	(0.46)	(2.89)	(8.62)	(4.87)
Saudi Arabia	−2.84^***^	−5.27^***^	4.24^***^	−3.56^***^	1.94^***^	0.95^***^	−4.33^***^	0.77^***^
	(3.81)	(4.88)	(3.03)	(7.43)	(3.53)	(5.41)	(3.32)	(9.86)
Iran	−0.35	−5.06^***^	−4.02^***^	−1.07^***^	−0.56	1.97^***^	12.1^***^	0.58^***^
	(0.35)	(7.58)	(4.18)	(4.22)	(1.27)	(6.56)	(9.32)	(5.04)
Thailand	−5.73^***^	−1.99^***^	−3.63^***^	−5.12^***^	−2.04^***^	−0.07	7.84^***^	2.23^***^
	(6.19)	(8.01)	(3.22)	(2.99)	(4.77)	(0.49)	(4.45)	(6.84)
Malaysia	−5.29^***^	−2.43^***^	−1.25	−5.26^***^	−0.05	0.46^***^	2.00^***^	1.34^***^
	(3.39)	(5.39)	(1.47)	(8.48)	(0.41)	(3.45)	(7.84)	(5.75)
Singapore	−2.16^***^	−1.48^***^	−2.83^***^	−0.62^***^	0.85^*^	−0.04	8.43^***^	0.45^***^
	(8.62)	(7.86)	(3.31)	(2.81)	(1.72)	(0.11)	(2.03)	(4.68)
Philippine	−6.29^***^	−2.09^***^	−3.98^**^	−4.58^***^	1.01^***^	0.95^***^	3.62^***^	0.35^***^
	(4.47)	(0.48)	(1.98)	(6.84)	(5.05)	(4.54)	(7.75)	(6.89)

*** *p < 0.01*,

** *p < 0.05, and*

* *p < 0.1*.

In Table 4, group-wise estimates of FMOLS reveal that trade liberalization exerts a significant and negative impact on infant mortality, and a positive and significant impact on life expectancy. The coefficient estimates reveal that a 1% increase in trade liberalization leads to a 2.000% decline in infant mortality and a 0.670% increase in life expectancy. Our study showed that trade liberalization contributes significantly to reducing the infant mortality rate and improves life expectancy in selected Asian economies. These findings are in line with previous studies (26, 33, 34). Trade liberalization can affect public health through technology transfer, more specifically importing processed foods and pharmaceutical products. Studies reveal that trade liberalization can influence public health through several pathways. Trade liberalization generates high income that contributes to economic development. Higher incomes help people buy nutrition, foods, prevention & treatment for disease, clean water access and raise living standards through better housing (35). Trade contributes significantly to technology transfer that influences public health through pharmaceutical R&D (36). It infers that public health can improve from developed technological innovation in pharmaceuticals and medical treatment through imports. Thus, the linkage between health outcomes and trade openness can activate through drugs and medical supplies, immunization rates, population, health expenditures, and income (33). Another benefit of trade liberalization is that it upsurges associations between economies and transfers knowledge regarding standard health practices and disease treatments, raising health programs and their related organization (33).

In terms of digitalization, internet use also has a significant and negative impact on infant mortality and produces a significant and positive impact on life expectancy. It is obvious from coefficient estimates that a 1% increase in the use of the internet tends to reduce infant mortality by 2.980% and increase life expectancy by 2.000%. The general meaning of these findings is that the internet can help promote public health in Asian economies. ICT, whether internet or mobile, is an important source to spread the knowledge related to different diseases and their remedies as well. Moreover, any new research related to the field of medical science done in developed economies can be disseminated to developing economies quickly with the help of the internet and cellular services. Internet and mobile technologies have connected people from all around the globe, and people in the developing world are now aware of the health care facilities available in the developed economies; thus, the demand for good health care facilities is also on the rise in developing economies (37). Over the last three decades, the growth of ICT has been witnessed, and it has exerted a positive impact on almost every sector of the economy, and health is not an exception. The availability of doctors has increased, and people can consult doctors online. Even the physical trip of a patient has been made easier by the ICT because they can make appointments online and save a lot of time. Online lectures and courses regarding medical care programs have become popular and support the speedy diffusion of medical-related knowledge. ICT can be utilized to raise the level of awareness, and health literacy among the people, which would be beneficial in improving the health status. Bukachi and Pakenham-Walsh (20) and Bankole (38) pointed out that hospital staff can use the ICT as a source of communication and a vital channel through which they can spread health care programs worldwide and form a global partnership against ailments.

The impacts of GDP and public health expenditure are negative and significant on infant mortality and positive and significant on life expectancy. The study also reports the findings of the DOLS model for confirming the robustness of the results. Almost all the coefficient estimates of DOLS models are quite similar to the findings of FMOLS models except public health expenditure in the life expectancy model.

In Table 5, the economy-wise findings of the FMOLS model reveal that trade has a significant and negative impact on infant mortality in all selected Asian economies except Iran; however, the magnitude is different in each economy. The coefficient estimates show that a 1% rise in bilateral trade results in reducing infant mortality by 6.33% in China, 9.14% in India, 1.01% in Japan, 3.28% in Indonesia, 3.45% in Turkey, 1.16% in the Republic of Korea, 2.84% in Saudi Arabia, 5.73% in Thailand, 5.29% in Malaysia, 2.16% in Singapore, and 6.29% in the Philippines. The findings demonstrate that bilateral trade exerts a positive and significant impact on life expectancy in China, India, Japan, Indonesia, Turkey, Saudi Arabia, Singapore, and the Philippines, and significant and negative impact on Thailand. The respective coefficient estimates demonstrate that in response to 1% increase in bilateral trade, life expectancy increases by 0.44% in China, 2.16% in India, 1.85% in Japan, 1.89% in Indonesia, 0.51% in Turkey, 1.94% in Saudi Arabia, 0.85% Singapore, and 1.01% in the Philippines, however, bilateral trade reduces life expectancy in Thailand by 2.04%. In the case of digitalization, the findings show that the internet has a negative and significant impact on the infant mortality rate in all selected Asian economies except India and the Republic of Korea. In terms of life expectancy, the internet exerts a significant and positive impact on life expectancy in all the selected economies except India, Singapore, and Thailand. The findings of the control variables reveal that GDP exerts a significant negative impact on infant mortality in eight of the economies and exerts a significant positive impact on life expectancy in 10 of the economies. However, health expenditures have a significant negative impact on infant mortality in 11 of the economies and a significant positive impact on life expectancy in 10 of the economies.

## Conclusion and Policy Implication

The impacts of trade and digitization are being explored in economic performance but are still unexploited in health. It is observed that trade and digitization should remain important tools, not just for economic purposes but also for improving health globally. Trade and digitization have been long debated at the center of policy. Aside from their impacts on economic growth, trade and digitization policies could leave an important mark on health. This study investigates the impact of trade and digitization on health in a sample of 12 Asian economies for a time period ranging from 1991 to 2018. The study used infant mortality and life expectancy variables to measure health. We applied the panel technique to investigate the regional and economy-wise estimates empirically.

Our findings show that trade and digitization play an imperative role in reducing infant mortality in Asian economies. Subsequently, trade and digitization have a significant positive impact on life expectancy, revealing in the long run. The findings of control variables demonstrate that GDP and health expenditure have a significant negative effect on infant mortality in the long run. The findings also infer that GDP and health expenditure play a significant role in enhancing life expectancy in Asian economies. The economy-wise results show that trade had a significant negative impact on infant mortality in China, India, Japan, Indonesia, the Republic of Korea, Saudi Arabia, Iran, Thailand, Malaysia, Singapore, and the Philippines. At the same time, the internet exerts a significant negative impact on infant mortality in China, Japan, Indonesia, Turkey, Iran, Saudi Arabia, Thailand, Malaysia, Singapore, Philippines. Trade exerts a significant positive impact on life expectancy in eight of the economies and exerts a significant negative influence in only one economy. However, the internet has a significant positive impact on life expectancy in just nine of the economies.

The findings of our empirical study propose some important policy implications. Governments should increase trade to obtain more financial resources to improve health outcomes. Authorities should include health professionals in future trade agreements and negotiations to help public health. Asian countries should remove import duties and trade barriers on health-related products. To improve the health of the Asian people, more free trade is needed, not less. Policymakers need to redesign economic policies which ensure inclusive ICT usage in society because it improves health by improving health literacy and information, health care services, and communication between patients and health care systems. Asian policymakers should increase ICT investments to improve population health. Policymakers and authorities should bring comprehensive trade and technology policies that reduce human suffering from COVID-pandemic.

Our study did not control other relevant healths determinants such as globalization, digitization, financial inclusion, energy consumption, and environmental pollution. Future studies could explore the links highlighted in this study by considering the sub-indicators of globalization, digitization, and health. Future studies should also consider financial globalization and ICT investments that affect health outcomes. It is hoped that future investigations will bring more contributions to technology innovation by considering health outcomes.

## Data Availability Statement

Publicly available datasets were analyzed in this study. This data can be found here: https://data.worldbank.org/indicator.

## Author Contributions

XinmZ: conceptualization and software. XinqZ: data curation and writing—original draft preparation. X-GY and FM: methodology, visualization, investigation, and writing—reviewing and editing. All authors contributed to the article and approved the submitted version.

## Funding

Research on the Difficulties and Countermeasures of Henan Rural Management Talents Shortage, Henan Philosophy and Social Science Fund (2018BJJ038); Restrictive Factors and Countermeasures for The Development of Cross-Border E-Commerce of Henan Agricultural Products, Henan Soft Science Project (202400410127); Research on Digital Economy Driving the High-quality Leapfrog Development of Henan Agriculture, 2021 Henan Universities Think Tank Project (2021-ZKYJ-03).

## Conflict of Interest

The authors declare that the research was conducted in the absence of any commercial or financial relationships that could be construed as a potential conflict of interest.

## Publisher's Note

All claims expressed in this article are solely those of the authors and do not necessarily represent those of their affiliated organizations, or those of the publisher, the editors and the reviewers. Any product that may be evaluated in this article, or claim that may be made by its manufacturer, is not guaranteed or endorsed by the publisher.
